# MLK3 is a newly identified microRNA-520b target that regulates liver cancer cell migration

**DOI:** 10.1371/journal.pone.0230716

**Published:** 2020-03-26

**Authors:** Fei Zhang, Yu Zhu, Shuhua Wu, Guodong Hou, Nianxiang Wu, Lirun Qian, Dong Yang

**Affiliations:** 1 Anhui Vocational College of Defense Technology, Lu'an, Anhui, China; 2 The Second Affiliated Hospital of Xi'an Jiaotong University, Xi'an, Shaanxi, China; 3 Anhui Provincial Children's Hospital, Hefei, Anhui, China; 4 Department of Hepatobiliary Surgery, Zhuji People's Hospital of Zhejiang Province, Zhuji, Zhejiang, China; Columbia University, UNITED STATES

## Abstract

The roles of microRNAs (miRNAs) in liver cancer have attracted much attention in recent years. In this study, we demonstrate that miR-520b is downregulated in MHCC-97H cells, a liver cancer cell line with high potential of metastasis, compared with MHCC-97L cells which has a low potential of metastasis. Furthermore, the enhanced expression of miR-520b could inhibit liver cancer cell migration, while silencing its expression resulted in increased migration. Mixed lineage kinase 3 (MLK3) was identified as a direct and functional new target of miR-520b. This regulation was also confirmed by luciferase reporter assays. In addition, our results showed that overexpression of the MLK3 expression partially reversed the effect of miR-520b on liver cancer cell migration, indicating that MLK3 contributes to the migration in liver cancer. The newly identified miR-520b/MLK3 axis partially elucidates the molecular mechanism of liver cancer cell migration and represents a new potential therapeutic target for liver cancer treatment.

## Introduction

Liver cancer is one of the most common malignancies and the second leading cause of cancer related death worldwide, particularly in Southeast Asia, including China, Korea and Japan [1]. Recently, next generation sequencing of liver cancer patients has revealed that tons of genes are deregulated during the progression of liver cancer, and that the dysregulation of these genes are closely correlated with liver cancer diagnosis and prognosis [2, 3]. It is important to identify the dysregulated genes in liver cancer and to investigate their roles in liver cancer carcinogenesis and progression.

MicroRNAs are a class of short noncoding RNAs, which are able to inhibit the expression of their target genes through binding with the 3’-untranslated region of target mRNA [4]. More and more studies has demonstrated that miRNAs can function as either tumor suppressors or oncogenes. Meanwhile, miRNAs are closely involved in tumor development, such as tumor proliferation, migration or metastasis [5, 6]. The dysregulated expression of many miRNAs have been reported before, such as miR-520b. The overexpression or downregulation of miR-520b has been reported in many cancers, such as glioma, lung cancer and ovarian cancer. MiR-520b is reported to suppress glioma progression by targeting MBD2 [7]. Meanwhile, MiR-520b restrains cell growth by targeting HDAC4 in lung cancer [8]. It is also reported that miR-520b promotes ovarian cancer growth [9]. Several studies revealed that miR-520b could inhibit liver cancer growth and functioned as a tumor suppressor miRNA [10, 11]. However, the effect of miR-520b on the migration of liver cancer is still uncertain.

MLK3, also known as MAP3K11, was first identified in 1994 [12]. MLK3 can phosphorylate and activate MAP2Ks, and then activate MAPKs. Overexpression of MLK3 was shown to induce transformation of NIH3T3 fibroblasts by activation of MEK signaling [13]. Recently, accumulating evidence showed that MLK3 may play important functions in tumor migration or invasion. It is reported that MLK3 promotes colorectal cancer invasion [14] MLK3 is also reported to drive invasion and migration in breast cancer cells [15]. However, the function of MLK3 in liver cancer needs further investigation.

In this study, our data demonstrated that miR-520b was downregulated in liver cancer cells. We also found that miR-520b inhibited the migration of liver cancer cells partially through targeting MLK3. Our results demonstrated the effect of miR-520b and MLK3 on the migration of liver cancer.

## Materials and methods

### Cell culture

L02, HepG2, MHCC-97L and MHCC-97Hcells were maintained in DMEM (Life Technologies, Inc., Gaithersburg, MD) supplemented with 10% fetal bovine serum (Life Technologies). Cultures were incubated at 37°C in a humidified atmosphere with 5% CO2. HepG2 cells used in this study were obtained from the ATCC (Manassas, VA, USA). Human HCC cell lines MHCC-97H and MHCC-97L were established in the Liver Cancer Institute of Fudan University, and these cells were generously endowed for our research [16]. Immortalized human liver cell line L-02 was from National Infrastructure of Cell line Resource (Shanghai, China).

### Quantitative reverse-transcription-PCR analysis

Extraction of total RNA of the cells and reverse transcription were carried out as described previously [17]. To quantify the expression miRNA-520b, RNA was isolated by Trizol method. Then, Reverse Transcriptase Reaction was processed (Promega, USA) according to manufacturer’s instructions. Quantitative real-time PCR was performed using the quantitative SYBR Green PCR kit (TaKaRa Bio, China). The primer of the miR-520b amplification is shown in S1 Table. GAPDH or U6 RNA was used as an endogenous control in our experiments.

### RNA interference

MiR-520b mimics(miR-520b), inhibitor(inh), miR-NC, inhibitor-NC(inhNC) were synthesized and purified by RiboBio (Guangzhou, China). The concentration of miR-520b, inh or inhNC was 100nM. Small interfering RNA targeting MLK3 (Si-MLK3) and negative control (Si-NC) were obtained from OriGene Technologies (Rockville, MD, USA). The concentration of Si-NC and Si-MLK3 was 100nM. Lipofecatmine 2000 (Invitrogen, USA) was used as transfection reagent as manufacture suggested. The experiments were done 48h after transfection unless clarified.

### Construction of plasmids

For the 3′UTR reporter vector, the 3′UTR sequence of MLK3 was cloned and inserted into a pGL3 plasmid (Promega, USA) (S1 Table). To introduce a deletion mutant of miR-520b seed sequence, we used a PCR based method to remove the part of seed region (S1 Table). The amplified full-length MLK3 from HepG2 cDNA was cloned into a pcDNA3 vector.

### Luciferase reporter assay

HepG2 Cells were transfected with plasmid containing the 3′UTR sequence of MLK3 (0.5μg), luciferase vector (0.5μg) and miR-520b (100nM) or miR-NC (100nM). Luciferase activities were measured 48h after transfection through a Dual-Luciferase Reporter Assay System (Promega, USA). Luciferase activity was normalized with the corresponding Renilla luciferase activity. Three independent experiments were repeated.

### Western blotting

Proteins were extracted by using a RIPA buffer. Polyvinylidene difluoride membranes were blocked in milk for 1h, then membranes were incubated with primary antibodies for 2 h. After incubated with secondary antibody 1h, the expression of protein was detected. The primary antibodies were anti-human MLK3(1:800, Invitrogen, USA), anti-phosphorylated c-Jun (1:1000, Cell signaling, USA) and anti-human β-actin(1:2000,Sigma, USA). Highly Cross-Adsorbed Secondary Antibody, HRP was from Invitrogen. Three independent experiments were repeated. Quantification of Western results was performed using ImageJ software.

### Wound healing experiment

Cells were seeded at 2× 10^5^ and were cultured overnight. After 24 h, when the transfected cells were grown to confluence and wounded by dragging a 200 μl pipette tip through the monolayer. Cell migration images were photographed when the scrape wound was introduced and 24 h or 36h after wounding through an inverted microscope. Three independent experiments were repeated.

### Migration assay

The migration ability of cells was measured as described previously [18]. In general, a polycarbonate membrane was used in all experiments. Cells were first added into the upper wells. The lower wells were filled with medium with FBS and PDGF. Cells migrated to lower wells were quantified. Three independent experiments were repeated.

### Statistics

Data were shown as mean ± SD. Data were analyzed by Student's *t* test or ANOVA. Differences were considered significant as *P*< 0.05.

## Results

### Downregulation of miR-520b in the liver cancer cells

**Fig 1:**
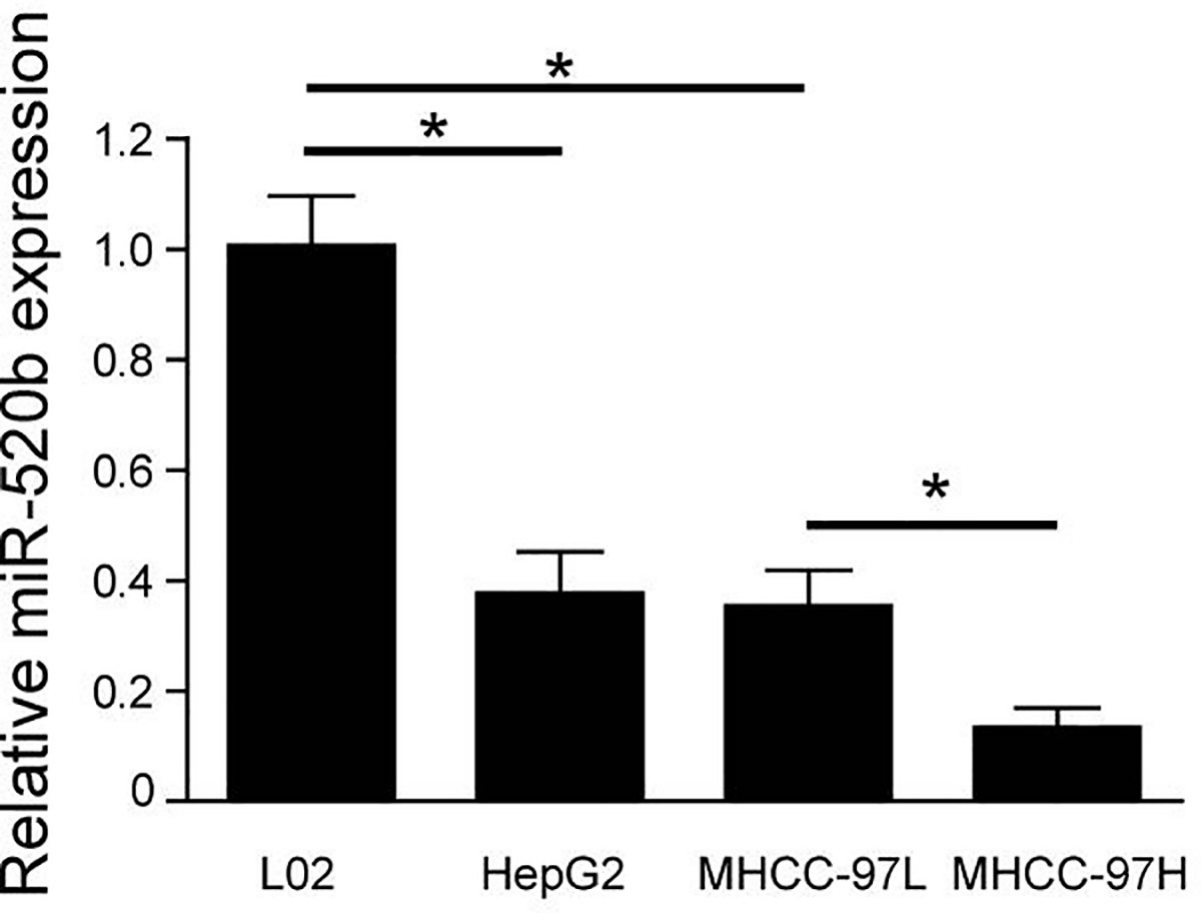
The relative expressions of miR-520b in L0-2, HepG2, MHCC-97H and MHCC-97L was measured by quantitative RT-PCR. U6 RNA was used as reference control. *P<0.05, data were shown as mean ± SD.

The downregulation of miR-520b was identified in liver cancer patients in a previous report [10]. To further confirm the function of miR-520b in liver cancer, we detected the expression of miR-520b in liver cancer cell lines. Our data showed that the expression levels of miR-520b were reduced in liver cancer cell lines relative to a normal liver cell line, L02 (Fig 1). Previous study showed that MHCC-97H cells have a high metastasis potential relative to MHCC-97L cells. Consistently, miR-520b expression was significantly decreased in MHCC-97H relative to MHCC-97L (Fig 1). Our results confirmed that the level of miR-520b was decreased in liver cancer cell lines. Meanwhile, downregulation of miR-520b may promote the migration of liver cancer cells.

### miR-520b overexpression decreases the number of invasive and migrated cells in liver cancer

**Fig 2:**
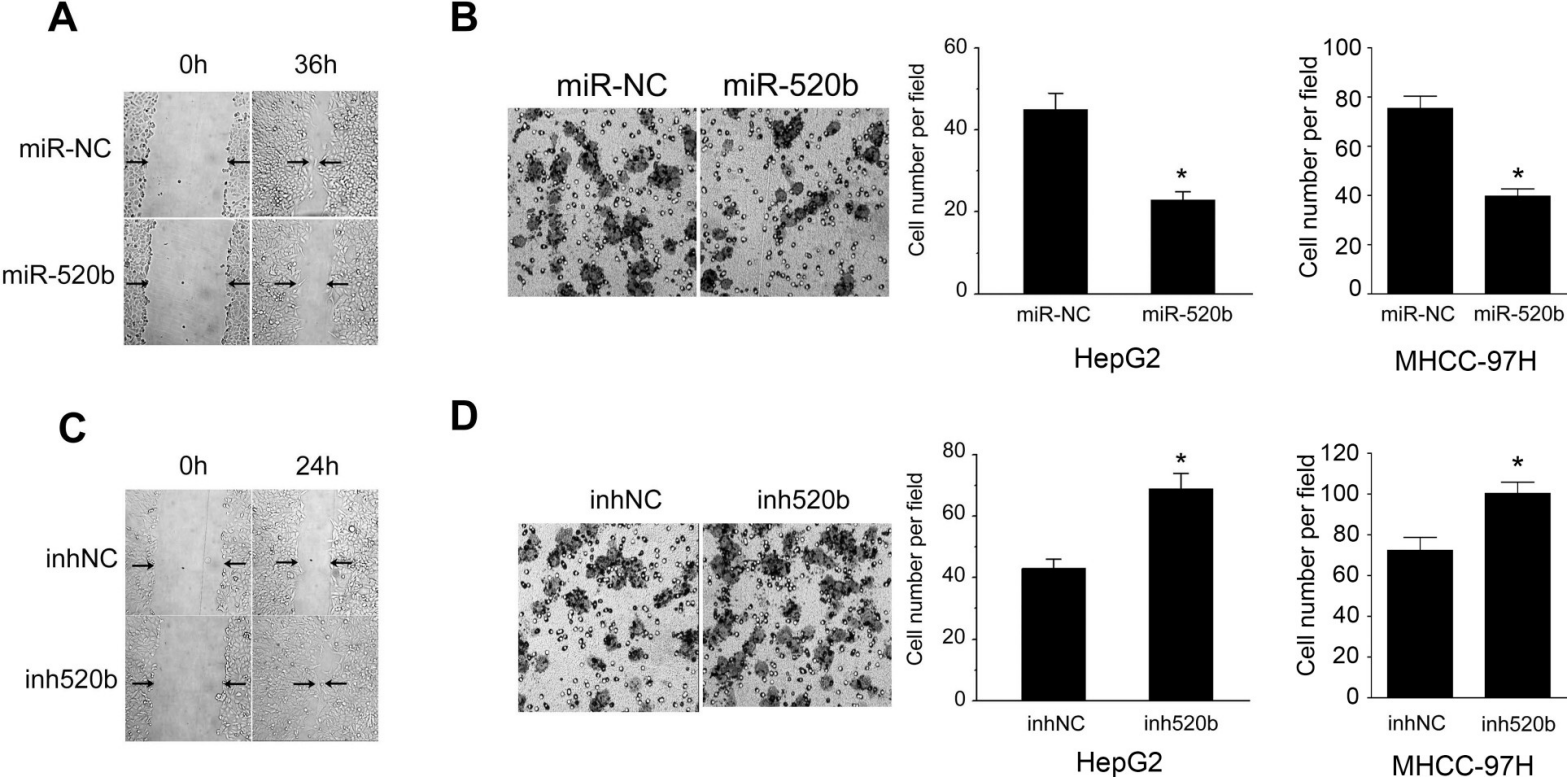
(A and C) A wound healing assay was performed on HepG2cells transfected with either miR-520b mimics (miR-520b) or a miR-520b inhibitor (ihn520b). miR-NC and inhNC are random RNAs used as control. The representative picture is showed. Black arrows showed the wound edge. (B and D) Migration assays was performed on HepG2 and MHCC-97H cells in the presence of either miR-520b or a miR-520b inhibitor (ihn520b). The number of cells in five randomly selected fields was calculated. *P<0.05, data were shown as mean ± SD.

To further study the biological effect of miR-520b on the migration ability in liver cancer, we measured the effect of miR-520b on the migration of HepG2 cells. Our data showed that overexpression of miR-520b significantly decrease the migration of liver cancer cells (Fig 2A and 2B). Meanwhile, miR-520b inhibitor reversed the effect of miR-520b (Fig 2C and Fig 2D). Moreover, our data also showed that similar effect of miR-520b or miR-520b inhibitor in MHCC-97H cells (Fig 2B and Fig 2D). Thus, our finding confirmed that miR-520b could inhibit the migration of liver cancer cells.

### MLK3 is a direct target of miR-520b

**Fig 3:**
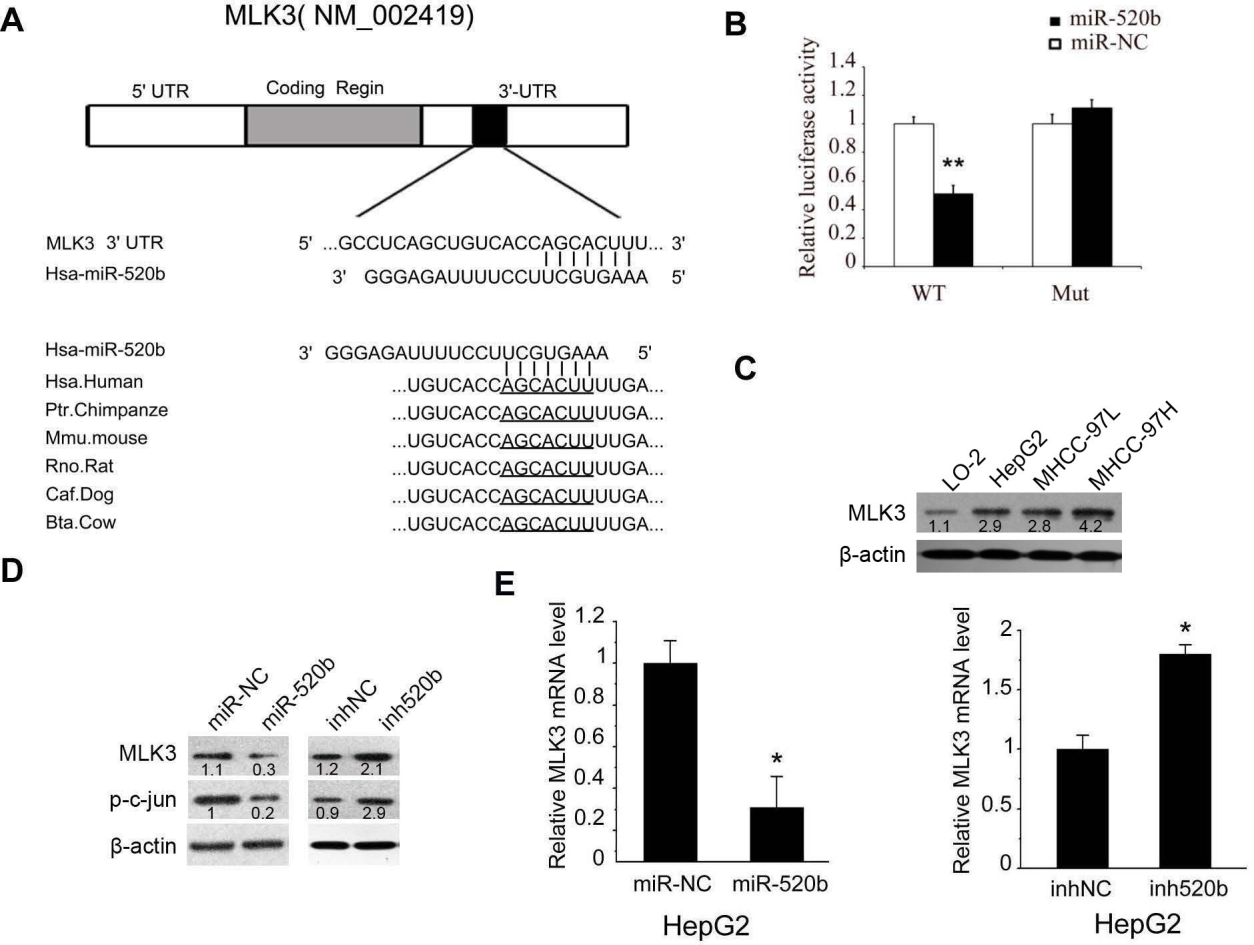
(A) Bioinformatics analysis (DIANA microT v3.0 algorithm) showed that miR-520b may direct target MLK3. (B) The effect of miR-520b on the activity of MLK3 3'UTR reporter was measured by luciferase reporter gene assay. (C) Immunoblot showed the protein level of MLK3 in cell lines. Quantification of Western results was performed using ImageJ software (D) Immunoblot showed that miR-520b reduced the level of MLK3 and p-c-jun and a miR-520b inhibitor (inh520b) increased the level of MLK3and p-c-jun in HepG2 cells. Quantification of Western results was performed using ImageJ software (E) Quantitative RT-PCR showed that miR-520b reduced the level of MLK3 and a miR-520b inhibitor (inh520b) increased the level of MLK3 in HepG2 cells. *P<0.05, data were shown as mean ± SD.

To further classify the underlying mechanism of miR-520b on migration, we try to identify the target genes of miR-520b. Through bioinformatics analysis, we found that MLK3 was predicted as target gene of miR-520b (Fig 3A).To confirm whether MLK3 is a direct target gene of miR-520b, we constructed luciferase reporter vectors containing the seed region of miR-520b from MLK3'UTR or a mutant-type vector (Four base pair seed sequence deleted) using PGL3 control vector, respectively (Fig 3B). We found that miR-520b could significantly reduce the luciferase activity of PGL3 vector with MLK3 3'UTR sequence but not the vector with mutant-type sequence. We then measured the protein level of MLK3 in L02, HepG2, MHCC-97L and MHCC-97H cells. Our data showed that the expression of MLK3 is upregulated in all the three liver cancer cell lines, which is correlated with the downregulation of miR-520b in those cells (Fig 3C). Moreover, our result also showed that overexpression of miR-520b significantly downregulated the expression of MLK3 in HepG2 cells at both mRNA and protein level (Fig 3D and Fig 3E). Furthermore, a specific miR-520b inhibitor upregulated the expression of MLK3 in HepG2 cells (Fig 3C and Fig 3D). Meanwhile, p-c-jun, one of the downstream target of MLK3, was also downregulated by miR-520b and upregulated by miR-520b inhibitor (Fig 3D). Both HBXIP and EGFR were reported as targets of miR-520b in other cancer cells. Here, we showed that miR-520b also could inhibit the expression of HBXIP and EGFR in HepG2 cells (S1A Fig). Our findings reveal that MLK3 is a newly identified miR-520b target in the liver cancer cells.

### Overexpression of MLK3 rescued the effect of miR-520b on liver cancer cell migration

**Fig 4:**
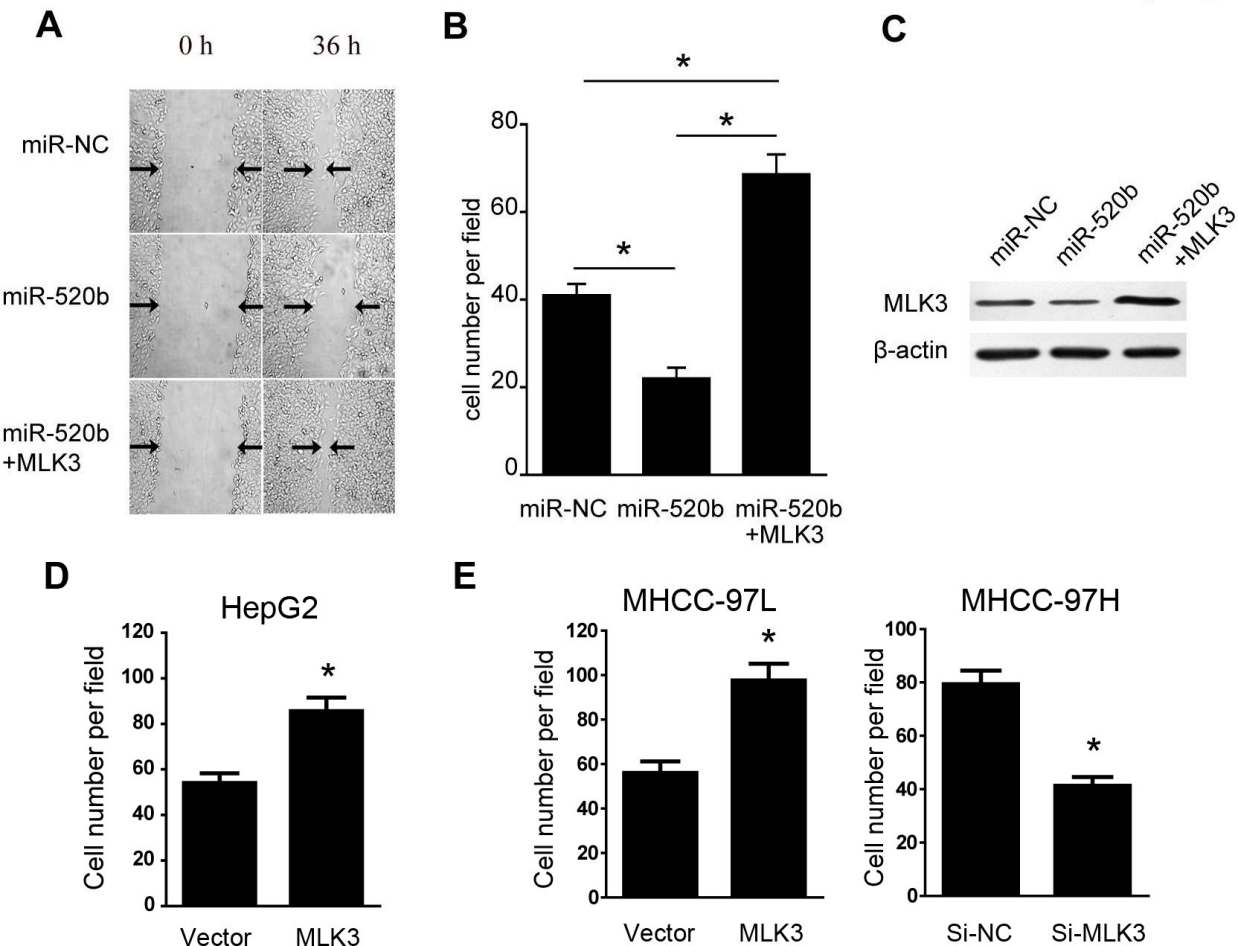
(A) Wound healing assay was performed in HepG2 cells transfected with either miR-520b or miR-520b together with a MLK3 overexpression vector. The representative picture is showed. Black arrows showed the wound edge. (B) Migration assays were performed on HepG2 cells transfected with either miR-520b or miR-520b together with a MLK3 overexpression vector. The number of cells in five randomly selected fields was calculated. (C) Immunoblot showed the protein level of MLK3. (D) Migration assays were performed on HepG2 cells transfected with either empty vector or a MLK3 overexpression vector. The number of cells in five randomly selected fields were showed.(E) Migration assays was performed on MHCC-97L cells transfected with MLK3 overexpression vector or on MUCC-97H cells transfected with siRNA targeting MLK3. The number of cells in five randomly selected fields was calculated. *P<0.05, data were shown as mean ± SD.

To further confirm whether miR-520b inhibited the migration of liver cancer cells through targeting MLK3, We cloned the sequence of MLK3 without the 3'-UTR region into an overexpressing plasmid and examined whether overexpression of MLK3 could reverse the effect of miR-520b in cell migration. As expected, we found that overexpression of MLK3 reversed the effect of miR-520b on cell migration (Fig 4A and 4B). We also confirmed the expression level of MLK3 in those cells and demonstrated that the expression of MLK3 was inhibited by miR-520b and was significantly increased after MLK3 overexpression (Fig 4C). Meanwhile, migration assay also showed that overexpression of MLK3 together with either miR-NC or miR-520b could increase the migration of HepG2 cells (S2 Fig). To further confirm the function of MLK3 in liver cancer cells, we overexpressed MLK3 in HepG2 cells and found that MLK3 significantly increased the migration of HepG2 cells (Fig 4D). Meanwhile, we also overexpressed MLK3 in MHCC-97L cells and silenced MLK3 in MHCC-97H cells. As expected, our result showed that MLK3 overexpression increased the migration of MHCC-97L cells and MLK3 knockdown decreased the migration of MHCC-97H cells, which further confirmed the function of MLK3 in the regulation of liver cancer migration (Fig 4E). Meanwhile, we also examined the effect of other target genes of miR-520b on liver cancer cell migration. We showed that silencing HBXIP or EGFR could also inhibit the migration of HepG2 cells, which suggests that the other target genes of miR-520b also contribute to the migration of liver cancer cells except MLK3. Our data reveal that miR-520b could reduce the migration of liver cancer cells partially through targeting MLK3.

## Discussion

Liver cancer is still one of the most common causes of cancer deaths in the world. The etiology and occurrence of liver cancer often related with the increasing incidence of HBV and HCV infection and accompanied with a poor prognosis. Although early stage surgery and diagnosis of liver cancer has improved, the worse prognosis and high risk of recurrence after surgery due to invasion and migration made the treatment of liver cancer challenging. Accumulating evidence indicated that the dysregulated expression of miRNAs play important roles during the progression of liver cancer. MiRNAs are involved in tumor initiation, migration and proliferation by regulating the expression of tumor suppressors or oncogenes. Although many miRNAs were dysregulated in liver cancer, the underlying mechanisms by which miRNAs regulate the processes of tumorigenesis and cancer progression are still largely unknown. In this work, we observed that miR-520b was significantly downregulated in a liver cancer cells with high metastasis potential. And then we demonstrated the miR-520b overexpression suppressed the migration of liver cancer cells. Meanwhile, we found that MLK3 was a newly identified target gene of miR-520b.

Many studies reported the role of miR-520b in human cancers. Most of the studies reported that miR-520b worked as a tumor suppressor in cancer. For example, it is reported that miR-520b inhibited proliferation of lung cancer, prostate cancer, glioma, head-neck cancer and colorectal cancer et al [7, 8, 19–22]. However, recently, the oncogenic role of miR-520b was also reported in ovarian cancer [9], which reveals that the effect of miR-520b may be context-dependent and needs further investigation in different tissues. Currently, accumulating evidences all indicate a tumor suppressor function in liver cancer [5, 10, 23]. Previous studies showed that miR-520b could inhibit growth or proliferation of liver cancer cells by target oncogenes, such as CCND1. Meanwhile, previous studies also reported that miR-520b could inhibit the migration of cancer cells in gastric cancer, breast cancer and Spinal Osteosarcoma Cells [24–26]. However, there is no report about the role of miR-520b on liver cancer migration or metastasis. Here we first demonstrated that miR-520b inhibited the migration and invasion of liver cancer cells *in vitro*. It is also reported that miR-520b could inhibit migration of gastric cancer cells [24], which is consistent with our results. Our data suggest a role of miR-520b on promotion of liver cancer migration.

MLK3 is widely reported as an oncogene in human cancers, especially in breast cancer [14, 15, 27, 28]. MLK3 is required during tumorigenesis and inhibition of MLK3 could block the oncogenic function of MLK3 in cancer cells. It is also reported that certain miR-125b and miR-199a-5p could target MLK3 and inhibit tumor migration or tumorigenesis [29, 30]. The function of MLK3 in liver cancer is still unclear. Our data showed that MLK3 is a direct target of miR-520b which may work as a tumor suppressor in liver cancer. Our also showed that overexpression of MLK3 rescued the effect of miR-520b on cell migration *in vitro*. Meanwhile, we believe that miR-520b regulates liver cancer cell migration not only through MLKs but also by HBXIP, EGFR or the other target genes. In our study, we mainly found that MLK3 is a new target of miR-520b and is involved in the promotion of liver cancer cells. Our findings support an oncogenic role of MLK3 in liver cancer, which needs further investigation.

## Conclusion

Our data support that miR-520b significantly suppressed the migration of liver cancer cells partially through targeting MLK3. Thus, the miR-520b/MLK3 axis may be a therapeutic target for controlling liver cancer migration.

## Supporting information

S1 Fig (A) Immunoblot showed that miR-520b reduced the level of HBXIP and EGFR on HepG2 cells. (B) Immunoblot showed the efficacy of silencing of HBXIP and EGFR. (C) Migration assays was performed on HepG2 cells transfected with siRNAs targeting HBXIP or EGFR.*P<0.05, data were shown as mean ± SD.
(PDF)

S2 Fig (A) Immunoblot showed the efficacy of overexpression and knockdown of MLK3 on HepG2 cells. (B) Migration assays was performed on HepG2 cells.*P<0.05, data were shown as mean ± SD. *P<0.05, data were shown as mean ± SD.
(PDF)

S3 Fig (PDF)

S4 Fig (PDF)

S5 Fig (PDF)

S1 Table Sequences of DNA and RNA oligonucleotides. (DOCX)

S1 Methods (DOCX)
