# Supplementary figures and images for: MLK3 is a newly identified microRNA-520b target that regulates liver cancer cell migration
Source: PLoS One. 2020 Mar 26;15(3):e0230716. doi: 10.1371/journal.pone.0230716 (PMC7098554; doi:10.1371/journal.pone.0230716)

**A**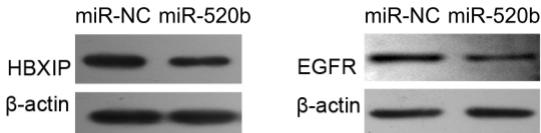**B**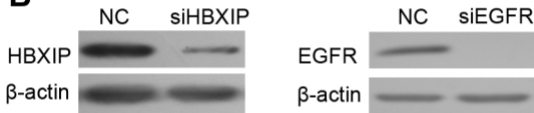**C**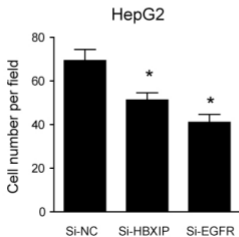

Supplement: S1 Fig — (A) Immunoblot showed that miR-520b reduced the level of HBXIP and EGFR on HepG2 cells. (B) Immunoblot showed the efficacy of silencing of HBXIP and EGFR. (C) Migration assays was performed on HepG2 cells transfected with siRNAs targeting HBXIP or EGFR.*P<0.05, data were shown as mean ± SD. (PDF) [file pone.0230716.s001.pdf]

**A****Zhang Fig S2**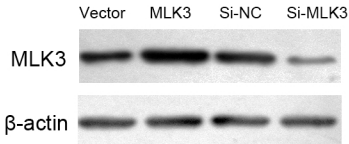**B**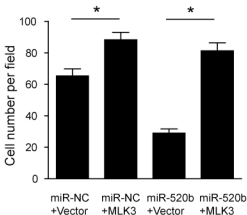

Supplement: S2 Fig — (A) Immunoblot showed the efficacy of overexpression and knockdown of MLK3 on HepG2 cells. (B) Migration assays was performed on HepG2 cells.*P<0.05, data were shown as mean ± SD. *P<0.05, data were shown as mean ± SD. (PDF) [file pone.0230716.s002.pdf]

MLK3

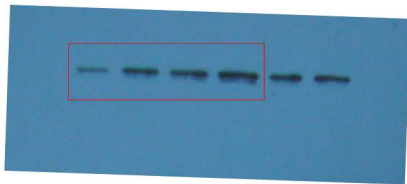

$\beta$ -actin

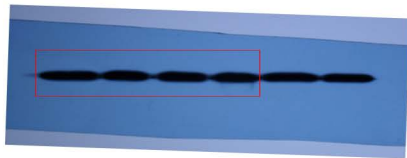

Supplement: S3 Fig — (PDF) [file pone.0230716.s003.pdf]

miR-520b  
inhibitor

MLK3

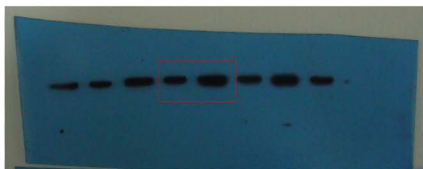

p-c-jun

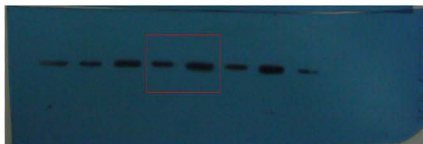

$\beta$ -actin

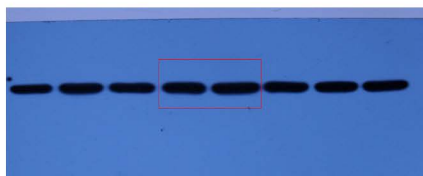

miR-520b

MLK3

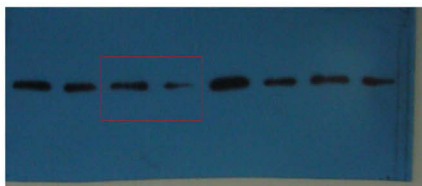

p-c-jun

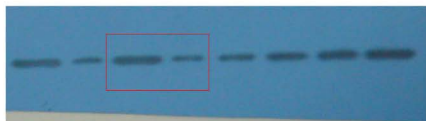

$\beta$ -actin

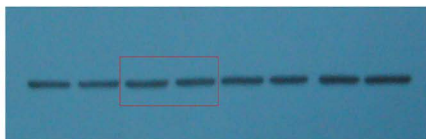

Supplement: S4 Fig — (PDF) [file pone.0230716.s004.pdf]

MLK3

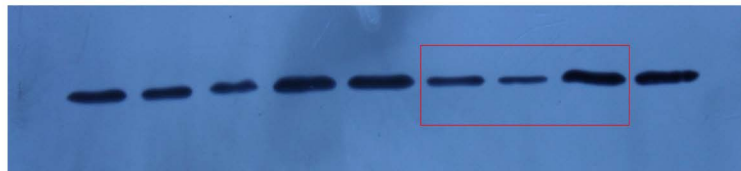

$\beta$ -actin

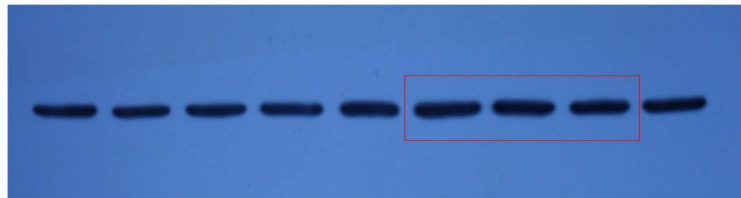

Supplement: S5 Fig — (PDF) [file pone.0230716.s005.pdf]
