# Supplementary material for: MLK3 is a newly identified microRNA-520b target that regulates liver cancer cell migration
Source: PLoS One. 2020 Mar 26;15(3):e0230716. doi: 10.1371/journal.pone.0230716 (PMC7098554; doi:10.1371/journal.pone.0230716)
Supplement: S1 Table — (DOCX) [file pone.0230716.s006.docx]

**Table S1.** Sequences of DNA and RNA oligonucleotides

| **Name** | **Sense Strand/Sense Primer (5′-3′)** | **Antisense Strand/Antisense Primer (5′-3′)** |
| --- | --- | --- |
| **MiRNA duplexes** | |  |
| miR-520b | AAAGUGCUUCCUUUUAGAGGG | CCCUCUACAGGGAAGCGCUUU |
| miR-NC | CGGUGAUAAUCUUUGUAGACG | CGUCUACAAAGAUUAUCACCG |
| **Primers for Cloning** | |  |
| pcDNA3-MLK3 | CCGGAATTCATGCGGAAGCGGGGCCTTGA | CCGCTCGAGTCAGCCTTCGCCCCGGAACT |
| MLK3 3′UTR-WT | CGTTCTAGAATTGCCTGCTGGGGTGATGC | GGGGGCCGGAGGAGGTCCATGCTCTAAGC |
| MLK3 3′UTR-Mut | CGGCCTCAGCTGTCACCGTTGCTTTTGACCA | CAACGGTGACAGCTGAGGCCGGCCCCTCTTC |
| **Primers for qRT-PCR** | |  |
| MLK3 | AGCAAACTCCGAGCAAGGGAC | GGCTAAACCAGAACTCAAGCGTG |
| miR-520b | AAAGTGCTTCCTTTTAGAGGG | GCGAGCACAGAATTAATACGAC |
| U6 | AGAGCCTGTGGTGTCCG | CATCTTCAAAGCACTTCCCT |
| GAPGH | GGATTTGGTCGTATTGG | GGAAGATGGTGATGGGAT |
