# Supplementary material for: MLK3 is a newly identified microRNA-520b target that regulates liver cancer cell migration
Source: PLoS One. 2020 Mar 26;15(3):e0230716. doi: 10.1371/journal.pone.0230716 (PMC7098554; doi:10.1371/journal.pone.0230716)
Supplement: S1 Methods — (DOCX) [file pone.0230716.s007.docx]

***RNA Interference***

The concentration of miR-520b, miR-NC, Si-NC, Si-HBXIP, Si-EGFR was 100nM. Small interfering RNA targeting HBXIP or EGFR (Si-HBXIP or Si-EGFR), and negative control (Si-NC) were obtained from Ruibo Biology (Guangzhou, China). The experiments were done 48h after transfection.

***Western Blotting***

The primary antibodies were anti-human HBXIP (1:1000, Abcam, UK) and anti-human EGFR (1:2000, Abcam, UK).
